# Advances in WRKY regulation of immune responses in medicinal plants

**DOI:** 10.3389/fpls.2025.1659732

**Published:** 2025-10-01

**Authors:** Xinying Zhong, Nana Chen, Hongwei Li, Yaxuan Wang, Ziyi Guo, Guiyuan Shi, Xingkai Zhan, Lin Li

**Affiliations:** Department of Cell Biology, Zunyi Medical University, Zunyi, Guizhou, China

**Keywords:** medicinal plants, immune responses, WRKY transcription factors, molecular mechanisms, immune regulation

## Abstract

Medicinal plants serve as a crucial source of traditional Chinese medicine and have garnered considerable attention due to their unique bioactive compounds and notable pharmacological properties. However, during natural growth, these plants are frequently susceptible to infection by various pathogenic microorganisms, pests and nematodes, leading to reduced yields and inconsistent accumulation of medicinal compounds, thereby significantly limiting their resource development and utilization. WRKY transcription factors (TFs) are central regulators of plant immunity that integrate pathogen-perception signals, coordinate signaling pathways, and transcriptionally control defense-gene expression**.** This review provides a systematic synthesis of current knowledge on the regulatory mechanisms of WRKY TFs in the immune responses of medicinal plants. Emphasis is placed on their roles in cellular metabolic regulation, activation of Mitogen-Activated Protein Kinase (MAPK) signaling pathways, integration of phytohormone signaling, and the biosynthesis of secondary metabolites. In addition, we highlight that WRKY TFs orchestrate immune responses at multiple levels through epigenetic mechanisms, including DNA methylation and histone modifications. Furthermore, it is proposed that transgenic approaches and Cut-Dip-Budding (CDB)-mediated transformation be integrated with gene editing technologies such as Clustered Regularly Interspaced Short Palindromic Repeats (CRISPR), in conjunction with artificial intelligence (AI)-assisted identification of key regulatory elements. This integrated strategy offers novel insights and theoretical support for establishing efficient immune regulatory networks and breeding disease-resistant medicinal plant varieties.

## 1 Introduction

Plants are naturally versatile and diverse, serving as essential sources of nutrients, pharmaceuticals, and chemical components (Owusu Adjei et al., 2021). Medicinal plants in particular contain natural compounds of important value in both traditional and modern medicine (Tanvir et al., 2024). They can be classified according to their medicinal parts, therapeutic effects, or main chemical constituents. For example, roots and rhizomes include *Panax ginseng* C.A.Mey. (Li et al., 2025a); flowers include *Lonicera japonica* Thunb. (Li et al., 2025b); leaves include *Ginkgo biloba* L. (Liu et al., 2022); fruits and seeds include *Lycium barbarum* L. (Shi et al., 2025); and whole herbs include *Leonurus japonicus* Houtt. (Wei et al., 2023). In terms of therapeutic effects, *Artemisia annua* L. is a typical antimalarial (Angupale et al., 2024), *Curcuma longa* L. is widely used for its notable anti-inflammatory effects (Tian et al., 2025), and *Astragalus membranaceus* Bunge is valued for its immunomodulatory potential (Wang et al., 2022a). These representative species are not only widely used in traditional medicine but also demonstrate significant immunological and therapeutic effects in modern pharmacological studies.

However, during their growth and development, medicinal plants are frequently attacked by viruses, pathogens, pests and nematodes (Han et al., 2025), which seriously affect their quality and medicinal value. Plants have developed sophisticated immune mechanisms in response to pathogen invasion (Yu et al., 2024). The plant immune system consists of two main layers of active defense. The first layer is triggered by the recognition of pathogen-associated molecular patterns (PAMPs) and host-derived damage-associated molecular patterns (DAMPs) by pattern recognition receptors (PRRs), which activate PAMP-triggered immunity (PTI). The second layer involves intracellular receptors called nucleotide-binding leucine-rich repeat receptors (NLRs), which trigger effector-triggered immunity (ETI) (Yuan et al., 2021). Although different receptors initiate PTI and ETI through separate signaling pathways, their downstream immune responses share significant overlap (Yuan et al., 2021). For example, PTI and ETI are closely linked through common signaling pathways like cell wall remodeling (Wan et al., 2021), activation of Mitogen-Activated Protein Kinase (MAPK) cascades, production of reactive oxygen species (ROS), and phytohormone signaling (Yang et al., 2025).

In plant immune response**,** transcription factors are central to the regulation of immune pathways (Xiang et al., 2025). The WRKY transcription factor (TF) family serves as a central regulator of plant immunity, modulating PTI and ETI responses either positively or negatively, while enhancing disease resistance through the regulation of secondary metabolite accumulation and epigenetic modifications (Chen et al., 2024a). Although the core components of plant immune mechanisms are largely conserved across species, medicinal plants display a unique characteristic: their immune signaling is closely integrated with secondary metabolite production, a feature that not only strengthens disease resistance but also directly affects their medicinal value (Li et al., 2025c; Zhao et al., 2023; Li et al., 2025d).

Currently, however, systematic understanding of WRKY transcription factors (TFs) in medicinal plant immunity remains limited. This study examines their regulatory role in innate immune responses and offers a foundation for enhancing disease resistance in medicinal plants.

## 2 From structure to function: the role of WRKY TFs in plant defense

WRKY TFs are a widely distributed family of plant-specific transcriptional regulators that recognize W-box sequences (*TTGACT/C*) in DNA and play crucial roles in diverse physiological processes, including seed germination, root development, stress adaptation, and immune defense (Wang et al., 2024a). In this paper, we specifically focus on their central role in mediating plant responses to pathogen invasion, emphasizing their key position as hubs within the defense regulatory network. WRKY proteins typically contain at least one WRKY domain, approximately 60 amino acids in length, featuring a highly conserved WRKYGQK motif at the N-terminus and a zinc finger motif at the C-terminus, both of which are essential for DNA binding (Zhang et al., 2023a). Based on structural characteristics, the WRKY family has been classified into three distinct groups. Group I contains two WRKY domains, each associated with a C_2_H_2_-type zinc finger motif at the C-terminus. Groups II and III possess a single WRKY domain, with C-terminal zinc finger motifs of the C_2_H_2_ and C_2_HC types, respectively (Rushton et al., 2010). These structural features enable WRKY TFs to recognize and bind specifically to W-box elements (*TTGACT/C*) in the promoters of downstream target genes, thereby precisely regulating gene expression and contributing to various biological processes (Li et al., 2024a), particularly those involved in plant immune responses (Liu et al., 2025).

During plant immune responses, WRKY TFs drive transcriptional reprogramming by recognizing and binding to W-box elements in the promoters of target genes, thereby activating key components of the salicylic acid (SA) signaling pathway, such as *NPR1/3*, *TGA*, and *PR1*, to enhance disease resistance (Li et al., 2024b). Studies have shown that RhWRKY30 directly binds to the W-box in the *RhCAD1* promoter, promoting lignin biosynthesis and enhancing resistance to *Botrytis cinerea* Pers. in *Rosa* spp (Li et al., 2024c). Similarly, class IIc WRKYs bind to the W-box in the *GhMKK2* promoter, thereby increasing *Gossypium hirsutum* L. resistance to *Fusarium oxysporum* Schltdl (Wang et al., 2022b). In addition, WRKY TFs often act synergistically with other TFs to regulate immune responses. For example, in *Rheum palmatum* L., WRKY and MYB factors synergistically activate genes involved in flavonoid biosynthesis, thereby promoting the accumulation of defensive secondary metabolites and enhancing both immune and chemical defenses (Zhou et al., 2022a).

Although WRKYs also participate in plant developmental processes, they establish relatively independent regulatory hubs during immune responses, with certain signaling pathways potentially shared with developmental networks (Liu, et al., 2024). This functional divergence enables the WRKY gene family to integrate multiple signals within complex transcriptional networks, thereby achieving precise reprogramming of immune-related gene expression and maintaining a central role in plant defense. To better illustrate these roles, we summarized the classification of WRKY TFs in medicinal plants and their immune mechanisms (**Table 1**).

**Table 1:** Classification of medicinal plant WRKY transcription factors and their mechanism of action in plant immunity.

Form	WRKY members	Medicinal plant	Machine	A pathogen	Diseases	Bibliography
I	*AtWRKY33*	*Arabidopsis thaliana*	Reduces the MeJA pathway defence gene *PDF1.2* expression; reduces the SA pathway defence gene *PR-1*. Binds to and activates the expression of the promoter of the secondary metabolite camalexin biosynthesis gene	*Alternaria* *brassicicola*; *Botrytis* *cinerea*	gray mold black spot disease	(Zheng et al., 2006)
	*AtWRKY55*		Enhancement of *PDF1.2* expression through regulation of *ORA59* promotes immune responses against soft rot disease	* Pectobacterium carotovorum* ssp. *carotovorum* (Pcc)	soft rot disease	(Kang et al., 2024)
	*BcWRKY33A*	*Brassica chinensis*	Direct activation of *BcMYB51-3* and downstream IGS biosynthetic gene expression	*Botrytis cinerea*	Gray mold	(Wang et al., 2022c)
	*SIWRKY3*	*Solanum lycopersicum*	Regulation of *TPK1b* affects the SA and ROS signalling pathways and negatively regulates resistance	*Botrytis cinerea*	Gray mold	(Luo et al., 2024)
	*SlWRKY22* *SlWRKY25*		Promotes stomatal closure and prevents pathogen invasion through stomata	*Pseudomonas syringae* pv. tomato	bacterial speck	(Ramos et al., 2023)
	*MdWRKY17*	*Malus domestica*	*MdMPK3-MdWRKY17-MdDMR6* pathway leads to apple disease susceptibility; *MdWRKY17* promotes SA degradation (*MdDMR6* is the promoter of the salicylic acid degradation gene)	*Colletotrichum fructicola*	Glomerella leaf spot (GLS)	(Shan et al., 2021b)
	*MdWRKY20*	*Malus domestica*	Binds to the promoter region of the immune-related gene *MdPR1* and activates its expression	*Fusarium solani*	apple replanting disease (ARD)	(Zhao et al., 2025)
	*FaWRKY25*	*Fragaria × ananassa* ‘Benihoppe’	Negative regulation of strawberry JA resistance signalling	*Botrytis cinerea*	gray mold disease	(Jia et al., 2020)
	*CaWRKY3*	*Capsicum annuum* L.	Induced by SA/MeJA/ETH; initiated defense genes (*CaPR1*, *CaNPR1*, *CaDEF1*)	*Ralstonia solanacearum*	bacterial wilt	(Hussain et al., 2024)
	*NbWRKY1*	*Nicotiana benthamiana*	Binds to *WHIRLY1* and inhibits *WHIRLY1* promoter activity, thereby deregulating WHIRLY1's negative regulation of *NbWRKY40*, *NbPR1*, and *NbPR2* and activating plant immune responses	*Geminivirus*	geminivirus infection	(Sun et al., 2023)
II	*PlWRKY65*	*Paeonia lactiflora*	Regulation of *PlPR* gene expression	*Alternaria tenuissima*	leaf spot disease	(Wang et al., 2020b)
	*RhWRKY13*	*Rosa* sp.	Inhibition of *RhCKX3*, *RhABI4* expression	*B. cinerea*	Gray mold	(Liu et al., 2023)
	*IiWRKY34*	*Isatis indigotica*	Positive regulation of lignin accumulation and stress tolerance	*Ralstonia solanacearum*	bacterial wilt	(Xiao et al., 2020)
	*SlWRKY8*	*Solanum lycopersicum*	Reduced *SlPR1* and *SlPR5* expression; up-regulated *SlPR1a1*, *SlPR7* expression	*Phytophthora infestans*	late blight; gray mold	(Gao et al., 2020)
	*SlWRKY16*		Suppression of SA (*PR-1*) and JA (*PI*) signalling pathway-related genes negatively regulates immune	*Meloidogyne javanica*	root-knot nematode worm disease	(Kumar et al., 2023)
	*AtWRKY75*	*Arabidopsis thaliana*	Interaction with JAZ8 derepresses the SA gene *ORA59* and positively regulates resistance	*Botrytis cinerea*; *Alternaria brassicicola*		(Chen et al., 2021d)
	*CaWRKY22b*	*Capsicum annuum*	Induction of HR cell death and H₂O₂ accumulation; activation of JA-responsive genes such as *CaDEF1*	*Ralstonia solanacearum*	bacterial wilt disease	(Shi et al., 2024)
	*AtWRKY8*	*Arabidopsis thaliana*.	Direct regulation of *ABI4*, *ACS6* and *ERF104* expression in ABA and ET immune signalling pathways	*Tobacco mosaic virus* China strain (TMV-cg)	Tobacco mosaic disease	(Chen et al., 2013)
	*LrWRKY39*	*Lilium regale* Wilson	Activation of SA signalling pathway-related genes enhances plant resistance to Phytophthora grey mold	*Botrytis cinerea*	gray mold disease	(Fu et al., 2022)
	*LrWRKY3*	*Lilium regale* Wilson	Involved in JA and SA-mediated signal transduction; up-regulated the expression levels of PRs and SODs; regulated defence-related genes	*Fusarium oxysporum*	Fusarium wilt	(Wang et al., 2022d)
	*PnWRKY9*	*Panax notoginseng* (Burk) F.H. Chen	Involved in MeJA signal transduction pathway to enhance disease resistance	*Fusarium solani*	root rot	(Zheng et al., 2022)
	*PnWRKY15*	*Panax notoginseng* (Burk) F.H. Chen	Up-regulation of resistance-related gene *PnOLP1*, activation of JA/SA signalling pathway	*Fusarium solani*	root rot	(Su et al., 2023b)
	*CaWRKY08-4*	*Capsicum annuum*	Activation of defence-related genes (1 *PR1*, 2 *PR4*, 1 pathogen-related gene)	*Phytophthora capsici*	Phytophthora blight	(Cheng et al., 2024)
	*CmWRKY15-1*	*Chrysanthemum morifolium*	Interacts with CmNPR1 to activate the expression of genes involved in downstream pathogenesis that enhance resistance through the SA pathway	*Puccinia horiana*	chrysanthemum white rust (CWR)	(Gao et al., 2022)
	*CsWRKY65*	*Citrus sinensis*	Up-regulates the expression of defence genes (e.g. ROS generation-related genes and disease-course-related protein genes), induces ROS accumulation and activates plant defence signalling pathways	*Penicillium digitatum*	Green mold	(Wang et al., 2021b)
	*AktWRKY11/18/21/31/47−2/51/65*	*Akebia trifoliata*	Involved in the regulation of pathogen-associated PTI/ETI immune responses	*Colletotrichum acutatum*	Anthracnose	(Wen et al., 2022)
III	*JcWRKY2*	*Jatropha curcas* L.	Regulation of SA mediated antioxidant enzymes	*Macrophomina* *phaseolina*	charcoal rot disease	(Dabi et al., 2020)
	*GhWRKY70*	*Gossypium hirsutum*	Positive regulation of the jasmonic acid (JA) signalling pathway	*Verticillium dahliae*	Verticillium wilt	(Zhang et al., 2023b)
	*PhWRKY30*	*Petunia hybrida*	Activation of SA biosynthesis gene *PhPAL2b* expression regulates disease resistance	*Tobacco rattle virus* (TRV) *Tobacco mosaic virus*(TMV)	Leaf curling; Chlorosis; Leaf mottling	(Wang et al., 2025)
	*JrWRKY21*	*Juglans regia* L.	*JrWRKY21* interacts with the transcriptional activator *JrPTI5L* to induce protein JrPR5L expression	*Colletotrichum gloeosporioides*	walnut anthracnose	(Zhou et al., 2022c)
	*JrWRKY4*		*JrWRKY4* was up-regulated by infestation, activated *JrSTH2L* expression and synergistically regulated immunity with *JrPHL8* and *JrVQ4* to enhance immunity	*Colletotrichum gloeosporioides*	anthrax of walnuts	(Mu et al., 2024)
	*ShWRKY81*	*Solanum habrochaites*	Activation of the SA signalling pathway to promote the expression of SA1 and downstream gene defence genes (e.g. *PR1*, *PR5*); enhancement of H₂O₂ accumulation and hypersensitivity reaction (HR) Activation of SA signalling pathway related genes and inhibition of JA signalling pathway related genes	*Oidium neolycopersici*	powdery mildew	(Wang et al., 2023b)
	*LrWRKY41a*	*Lilium regale* Wilson	Activation of SA signalling pathway related genes and inhibition of JA signalling pathway related genes	*Botrytis cinerea*	gray mold	(Fu et al., 2022)
	*CaWRKY70*	*Cicer arietinum* L*. *	Negative regulation inhibits *CaMPK9-CaWRKY40* signal transduction; inhibits defence genes *PR1*, *PR5*	*Fusarium oxysporum* f. sp. *ciceri* Race1 (Foc1)	wilt	(Chakraborty et al., 2020)
	*MiWRKY53*	*Morus indica*	Mediation of defence pathways through SA, including activation of the SA signalling pathway, upregulation of *PR-1* gene expression	*Pseudomonas syringae* PstDC3000	bacterial speck disease	(Negi et al., 2021)
	*CaWRKY01-10*	*Capsicum annuum* L.	Activation of the same 4 defence-related genes (1 *PR1*, 2 *PR4* and 1 pathogen-related gene)	*Phytophthora capsici*	Phytophthora blight	(Cheng et al., 2024)
	*OscWRKY1*	*Ocimum sanctum *	Binding the promoters of key genes of the phenylpropane pathway (e.g. *PAL* and *C4H*) in *Arabidopsis thaliana* activates their expression and increases the content of rosmarinic acid, thereby enhancing disease resistance.	*Pseudomonas syringae* pv. Tomato DC3000	bacterial disease	(Joshi et al., 2022)

## 3 Mechanisms of WRKY-mediated immune responses in medicinal plants

WRKY TFs serve as central hubs in the immunoregulatory networks of medicinal plants, synergistically modulating multiple layers of defense, including structural barrier reinforcement, oxidative stress mitigation, signal transduction, and metabolic defenses in response to pathogen attack (Wang et al., 2024a; Chen et al., 2025a). In *Arabidopsis thaliana* (L.) Heynh., WRKY research has primarily elucidated their conserved roles in plant immunity (Wang et al., 2024a). By contrast, in medicinal plants such as *Panax notoginseng* (Burk.) F.H.Chen (Su et al., 2024), *Gastrodia elata* Bl. f. *glauca* S. Chow (Wang et al., 2020a), and *Salvia miltiorrhiza* Bunge (Yu et al., 2025), WRKY factors more prominently mediate the crosstalk between immune signaling networks and secondary metabolic pathways (Li et al., 2025c). Their downstream MAPK cascades and hormone signaling pathways exhibit species-specific responses, thereby tightly coupling defense reactions with the biosynthesis of medicinally active metabolites. This integration represents the defining feature that distinguishes immune research in medicinal plants from studies in other plant systems (Li et al., 2025c, e).

Mechanistically, WRKY TFs upregulate genes involved in lignin biosynthesis, thereby enhancing cell wall-mediated defense. They also modulate antioxidant enzyme systems to alleviate pathogen-induced ROS accumulation and reduce oxidative damage. At the level of signal transduction, WRKY TFs often act synergistically with the MAPK cascade to promote the activation of defense-related genes. In the hormonal signaling network, WRKYs finely regulate immune responses by interacting with key phytohormones, including jasmonic acid (JA), SA, and ethylene (Wang et al., 2024a; Javed and Gao, 2023). For example, PnWRKY9 in *Panax notoginseng* activates the JA signaling pathway, enhances the expression of the antimicrobial peptide gene *PnDEFL1*, and increases resistance to *Fusarium solani* (Zheng et al., 2022). Meanwhile, WRKY TFs have also been shown to directly or indirectly regulate genes involved in the biosynthesis of key secondary metabolites, such as flavonoids, terpenoids, and alkaloids, thereby enhancing metabolic defenses. For instance, *EbWRKY30, EbWRKY31, and EbWRKY44* are co-expressed with structural genes involved in flavonoid biosynthesis in *Erigeron breviscapus* (Vaniot) Hand.-Mazz., leading to enhanced antioxidant capacity and disease resistance (Song et al., 2024d).

**Figure 1:**
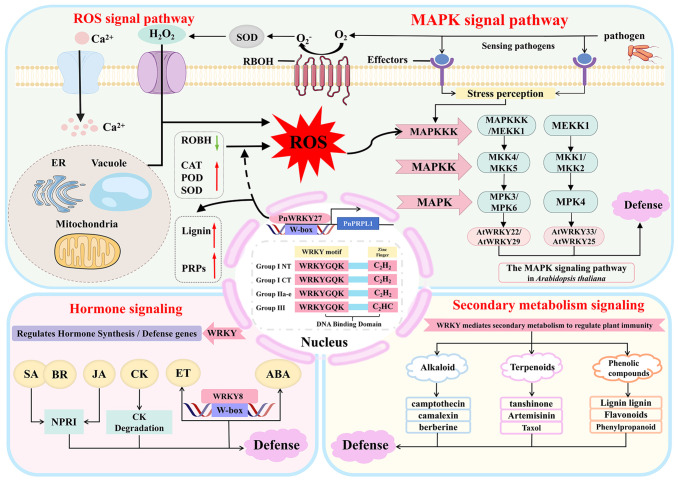
Regulatory mechanisms of WRKY transcription factors in the immune signaling network of medicinal plants. Dashed lines are speculative paths, solid lines are experimentally supported pathways; arrows indicate activation (→) or inhibition (⊣); ER, endoplasmic reticulum; ROS, Reactive Oxygen Species; CAT, Catalase; POD, Peroxidase; SOD, Superoxide Dismutase; RBOH, Respiratory Burst Oxidase Homolog; MAPK, Mitogen-Activated Protein Kinase; MAPKK/MKK, Mitogen-Activated Protein Kinase Kinase; MAPKKK, Mitogen-Activated Protein Kinase Kinase Kinase; MEKK1, Mitogen-Activated Protein Kinase Kinase Kinase 1; MKK2, Mitogen-Activated Protein Kinase Kinase 2; MPK3, Mitogen-Activated Protein Kinase 3; MPK4, Mitogen-Activated Protein Kinase 4; PRPs, Pathogenesis-Related Proteins; SA, Salicylic Acid; JA, Jasmonic Acid; ET-Ethylene; ABA, Abscisic Acid; CK, Cytokinin; BR, Brassinosteroids.

Collectively, these studies demonstrate that WRKY factors play a central, multidimensional, and synergistic role in the immune network of medicinal plants, providing novel insights for the molecular breeding of highly resistant medicinal plant varieties. These regulatory mechanisms are further illustrated in the immune signaling network of medicinal plants (**Figure 1**).

### 3.1 WRKY mediates the regulation of medicinal plant immune responses at the intracellular physiological and biochemical level

#### 3.1.1 WRKY mediates lignin regulation of medicinal plant immune responses

In various medicinal plants, WRKY TFs have been shown to play a crucial role in lignin biosynthesis and pathogen defense. As a major component of plant secondary cell walls (Ma, 2024), lignin serves as a key marker of bio-induced immune responses (Xiao et al., 2022). It constitutes the first line of defense against pathogen invasion by interacting with cellulose and other cell wall components to enhance mechanical strength and reduce permeability (Ma, 2024). WRKY TFs contribute to plant immune responses by regulating the phenylpropanoid pathway, thereby promoting lignin accumulation (Xiao et al., 2023). For example, WRKY11 in *Lilium regale Wilson* enhances resistance to *usarium oxysporum* by suppressing the expression of the *LrCel1* gene, thereby reducing cellulase activity and increasing lignin content (Chen et al., 2025b). SmWRKY40 in *Salvia miltiorrhiza* and NtWRKY28 in *Nicotiana tabacum* L. are both involved in regulating lignin biosynthesis. Studies have shown that SmWRKY40 is associated with phenylpropanoid metabolism and the stability of root cellular structures (Yu et al., 2025), while NtWRKY28 upregulates the expression of key lignin biosynthetic genes (such as *CAD*, *CCR*, and *HCT*) and promotes the accumulation of defense-related metabolites, including lignin and flavonoids, thereby significantly enhancing resistance to aphid infestation (Chu et al., 2025). Overall, WRKY TFs play a central role in immune response by promoting lignin biosynthesis, reinforcing mechanical barriers, and coordinating the regulation of secondary metabolic pathways, thereby enhancing environmental adaptability and stress tolerance.

#### 3.1.2 WRKY-mediated regulation of antioxidant enzymes in medicinal plant immunity

Upon pathogen attack, plants not only establish a first line of defense by strengthening cell wall mechanical properties, but also rapidly activate an immune signaling network centered around ROS (Haghpanah et al., 2025). ROS function as key signaling molecules that initiate defense pathways during early immune responses, but their excessive accumulation induces oxidative stress and leads to cellular damage. To maintain ROS balance, plants regulate the expression of antioxidant enzymes (including superoxide dismutase (SOD), catalase (CAT), and peroxidase (POD)) through WRKY TFs, thereby scavenging excess ROS and enhancing disease resistance.

*PnWRKY27* in *Panax notoginseng* specifically binds to the *PnPRPL1* promoter, promoting PnPRPL1 protein synthesis, which in turn regulates the expression and enzymatic activities of antioxidant enzymes (CAT, POD, and SOD), maintains intracellular ROS homeostasis, and enhances resistance to Fusarium root rot (Su et al., 2024). Overexpression of *CsWRKY25* in *Citrus* spp. and heterologous expression of SpWRKY1 in *Nicotiana tabacum* upregulate the transcription and enzymatic activity of antioxidant enzymes such as SOD, CAT, and POD, promote ROS scavenging, and activate phosphorylation-related signaling pathways, thereby enhancing plant resistance to pathogens (Wang et al., 2021a; Li et al., 2015a). In *Chrysanthemum morifolium*, constitutive overexpression of *CmWRKY48* markedly suppressed aphid population growth, indicating its pivotal role in aphid resistance (Li et al., 2015b).

It is noteworthy that not all WRKY TFs contribute to positive regulation of plant defense. For example, overexpression of *CaWRKY20* suppressed the transcription of ROS scavenging-related enzyme genes (*CaCAT*, *CaPOD*, *and CaSOD*), thereby reducing the ROS scavenging capacity of cells, leading to excessive accumulation of H_2_O_2_, and weakening the resistance of plants to *Colletotrichum* spp (Li et al., 2025f). In addition, overexpression of *CmWRKY53* suppressed POD gene expression, thereby increasing *Chrysanthemum* susceptibility to aphids and offering a molecular basis for its susceptibility mechanism (Zhang et al., 2020).

At the same time, ROS functions as an upstream signal in the MAPK cascade, triggering the phosphorylation and activation of MPK3/MPK6 (mitogen-activated protein kinase 3/6) and other kinases. WRKY TFs regulate ROS homeostasis and act as MAPK pathway targets, linking signal perception to gene expression and mediating plant immune responses.

### 3.2 WRKY-mediated protein kinase MAPK cascade pathway regulates immune responses in medicinal plants

The MAPK cascade response, which consists of three layers of kinases: Mitogen-Activated Protein Kinase Kinase Kinase (MAPKKK), Mitogen-Activated Protein Kinase Kinase (MAPKK), and MAPK, is one of the immune signaling pathways that is rapidly activated by plants upon sensing pathogens (Wu and Wang, 2024). Once activated, MAPKs regulate the expression of specific downstream immune-related genes by modulating the activity of various TFs, including WRKY, MYB, and ERF (Zhang and Zhang, 2022). Among these, WRKY TFs have been identified as primary targets of MAPKs and play a central role in immune signaling by bridging signal transduction with downstream gene expression (Laflamme, 2023).

In *Arabidopsis thaliana*, PAMP signaling activates two distinct MAPK-WRKY pathways. One is the MEKK1 (mitogen-activated protein kinase kinase kinase 1)-MKK4/5 (mitogen-activated protein kinase kinase 4/5)-MPK3/6 cascade, leading to the activation of WRKY22 and WRKY29, which enhances plant resistance to pathogens (Asai et al., 2002). The other is the MEKK1-MKK1/2-MPK4 pathway, in which MPK4 phosphorylates the transcriptional regulatory protein MAP kinase substrate 1 (MKS1). MKS1 subsequently regulates its interacting partner WRKY33, which negatively regulates the plant immune response to prevent excessive activation. However, under certain pathogen stresses, such as infection by *Pseudomonas* spp., WRKY33 remains active. In such cases, WRKY33 can mediate the expression of downstream defense-related genes (Kong et al., 2012). Additionally, WRKY33 is activated by MPK3/6-mediated phosphorylation, which promotes the expression of camalexin biosynthesis genes in coordination with ERF1, thereby enhancing Arabidopsis resistance to *Botrytis cinerea* (Zhou et al., 2022b).

This mechanism has also been observed in other plant species. In *Nicotiana tabacum*, *NtWRKY4*, *NtWRKY6*, and *NtWRKY10* interact with the MAPK cascade and positively regulate immune responses against whitefly infestation (Yao et al., 2020a). Similarly, *PnWRKY35* from *Panax notoginseng* has been shown to activate MAPK signaling and enhance disease resistance when ectopically expressed in *Nicotiana tabacum* (Li et al., 2025a). However, in *Malus domestica*, activation of the MKK4-MPK3-WRKY17 signaling pathway reduces SA levels, resulting in increased susceptibility to Glomerella leaf spot, indicating that this MAPK-WRKY module may function as a negative regulator in plant immunity (Shan et al., 2021).

In summary, MAPK-WRKY signaling modules play widespread roles in pathogen recognition and immune regulation across diverse plant species and can function in both positive and negative regulation, emphasizing the complexity and precise modulation of plant immune networks required for maintaining dynamic homeostasis.

### 3.3 WRKY mediates hormonal regulation of immune responses in medicinal plants

**Figure 2:**
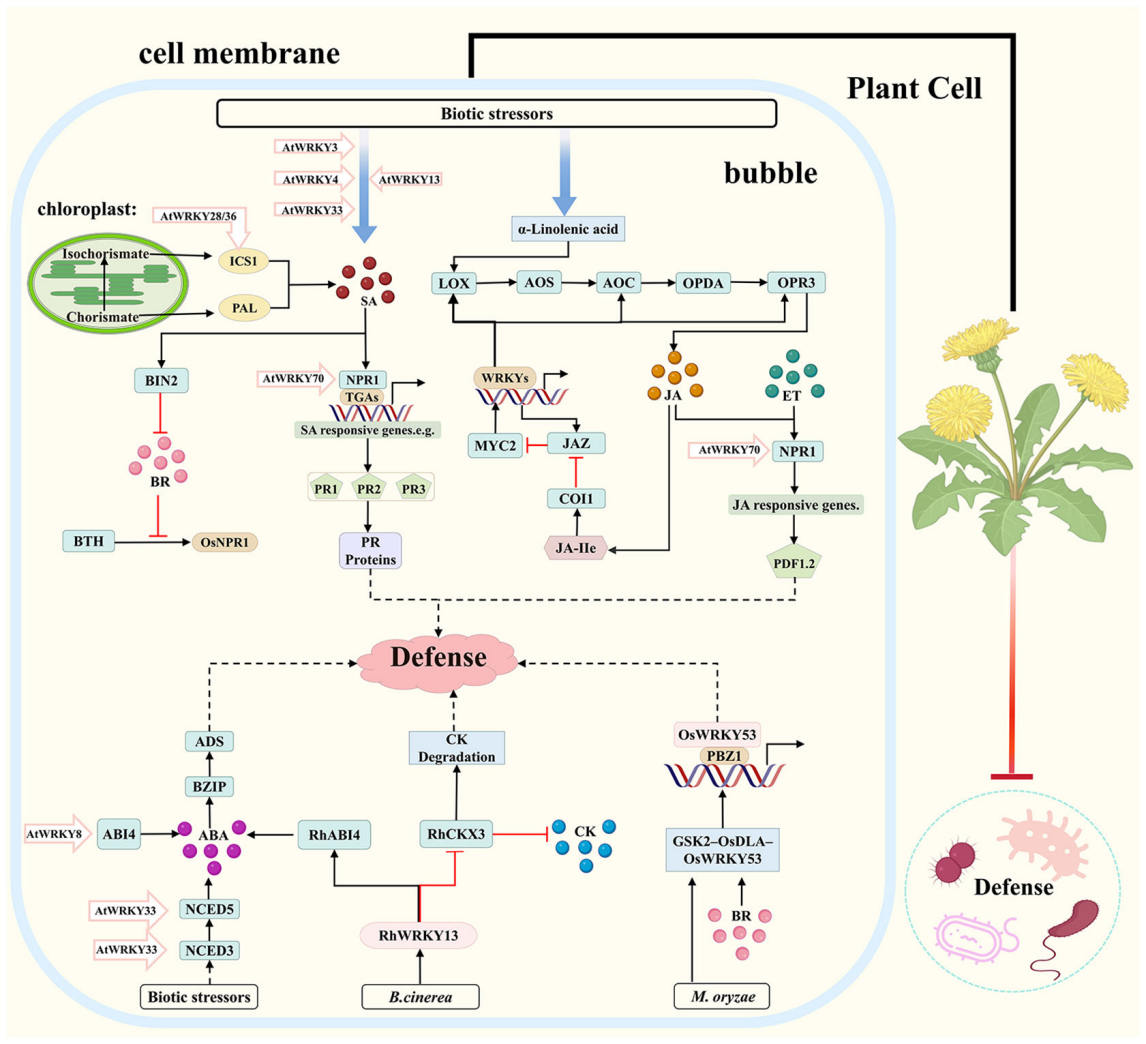
Map of WRKY-regulated hormonal immune defense mechanisms in medicinal plants. Dashed lines are speculative paths, solid lines are experimentally supported pathways; arrows indicate activation (→) or inhibition (⊣); JA, Jasmonic Acid; SA, Salicylic Acid; ET, Ethylene; ABA, Abscisic Acid; CK, Cytokinin; BR, Brassinosteroid; LOX, Lipoxygenase; AOS, Allene Oxide Synthase; AOC, Allene Oxide Cyclase; OPDA, 12-oxo-phytodienoic acid; OPR3, 12-oxo-phytodienoic acid reductase 3; MYC2, bHLH Transcription Factor MYC2; JAZ, Jasmonate ZIM, domain; COI1, Coronatine Insensitive 1; JAIIe, JA, Ile (Jasmonoyl, Isoleucine); ICSI, Isochorismate Synthase I; PAL, Phenylalanine Ammonia, Lyase; NPR1, Nonexpressor of Pathogenesis, Related genes 1; OsNPR1, Oryza sativa NPR1; TGAs, TGA transcription factors; BIN2, Brassinosteroid-Insensitive 2; BTH, Benzothiadiazole; PR Proteins, Pathogenesis-Related Proteins; ADS, Amorpha-4,11-diene Synthase; BZIP, Basic Leucine Zipper; ABI4, ABA-Insensitive 4; NCED5/3, 9-cis-Epoxycarotenoid Dioxygenase 3/5; RhCKX3, Rosa Cytokinin Oxidase/Dehydrogenase 3; RhABI4, Rosa ABA, Insensitive 4; GSK2-OsDLA-OsWRKY53, Glycogen Synthase Kinase2-DLA-WRKY53; PBZ1, Pathogenesis-related protein PBZ1; RhWRKY13, Rosa WRKY13; AtWRKY, Arabidopsis WRKY.

Upon pathogen attack, the MAPK cascade is rapidly activated, leading to the phosphorylation and activation of WRKY TFs, which serve as key hubs that link early pathogen recognition to downstream immune responses. WRKY TFs form a core regulatory network for disease resistance by modulating antagonistic and synergistic interactions among immune-related hormones such as SA, JA, and ET, and by coordinating signaling pathways involving gibberellin (GA), brassinosteroids (BR), auxin (IAA), and strigolactones (SL) to enhance precise pathogen recognition and improve environmental adaptability in plants (Wani et al., 2021; Wang et al., 2023a; Goyal et al., 2023). This complex regulatory framework is depicted in the map of WRKY-regulated hormonal immune defense mechanisms in medicinal plants (**Figure 2**).

In the model plant *Arabidopsis thaliana*, WRKY70 serves as a pivotal node in the antagonistic regulation between SA and JA/ET, promoting the expression of SA-dependent resistance genes such as PR proteins while repressing genes in the JA/ET pathway (Jiang et al., 2015). In addition, WRKY25, WRKY33, WRKY11, and WRKY17 also participate in this crosstalk regulation (Li et al., 2004; Journot-Catalino et al., 2006; Zheng et al., 2007). In *Nicotiana attenuata*, NaWRKY3 functions as a key transcriptional regulator during *Alternaria alternata* infection, activating jasmonate biosynthetic genes (*NaLOX3*), ethylene biosynthetic genes (*NaACS1*, *NaACO1*), ROS-generating genes (*NaRbohD*), and defense-related secondary metabolite genes (*NaF6’H1*, *NaBBL28*), thereby enhancing antifungal immunity through the integration of hormonal, oxidative, and metabolic responses (Xu et al., 2023). These findings provide important insights into elucidating the immune mechanisms of medicinal plants.

In medicinal plants, WRKY TFs likewise serve as central hubs of hormone regulatory networks. For example, in *Pinus massoniana*, exogenous signaling molecules (MeJA, SA, etc.) rapidly induce the expression of *PmWRKY31*, which regulates *PmLp8* to activate downstream hormone signaling and terpene biosynthesis genes, thereby elevating endogenous levels of MeJA, GA, SA, and abscisic acid (ABA), promoting the accumulation of terpenes and volatiles, and ultimately enhancing resistance to *Dendrolimus punctatus* (Chen et al., 2021a). In *Panax ginseng*, *PnWRKY15* synergistically regulates the SA and JA pathways and activates the resistance gene *PnOLP1*, thereby strengthening resistance to root rot disease (Su et al., 2023);Similarly, in *Paeonia lactiflora*, *PlWRKY65* induces the expression of defense genes such as *PlPR1* and enhances systemic immune responses, possibly through the coordination of SA-JA signaling (Wang et al., 2020b).

In addition to the backbone hormones such as SA, JA, and ET, WRKY TFs are also widely involved in the defense regulation of hormones including ABA, cytokinin (CK), and BR. In *Nicotiana attenuata*, NaWRKY70 directly activates the biosynthetic genes of JA (*NaAOS*, *NaJAR4*) and ABA (*NaNCED1*, *NaXD1-like*), while simultaneously promoting the accumulation of *NaF6’H1*-mediated defense metabolites, scopoletin and scopolin, thereby rapidly initiating resistance against *Alternaria alternata* (Song and Wu, 2024b; Song and Wu, 2024c). Several studies have demonstrated that ABA and CK often act antagonistically in plant immunity. In *Rosa hybrida*, ABA enhances susceptibility, whereas exogenous CK increases resistance. Mechanistically, RhWRKY13 strengthens defense against grey mould by repressing the CK-degrading gene *RhCKX3* and the ABA-responsive factor *RhABI4*, thereby enhancing CK signaling and suppressing ABA responses (Liu et al., 2023). Moreover, BR was also shown to improve rose petal resistance to *Botrytis cinerea*, possibly by regulating the expression of TFs such as WRKY, together with cell wall receptors and hormone signaling-related genes (Liu et al., 2018).

In summary, WRKY TFs, as key regulatory nodes of hormone signaling, not only coordinate synergism and antagonism among immune hormones, but also construct an efficient and dynamic immune network by regulating defense genes and metabolic pathways to help medicinal plants to cope with the complex pathogen environment.

### 3.4 WRKY mediates secondary metabolite synthesis to regulate immune responses in medicinal plants

In recent years, plant immunity research has gradually expanded from traditional focuses on pathogen recognition and signal transduction to defense strategies centering on secondary metabolite-mediated immunity. These metabolites not only have strong toxic inhibitory effects on pathogenic microorganisms, but also serve as important barriers for plants against multiple classes of stresses by modulating insect feeding behavior and nematode movement. Pathogen, pest or nematode infestation induces key TFs such as WRKY, MYB, bHLH, etc., which regulate multiple metabolic pathways and promote the accumulation of multiple classes of defensive metabolites such as alkaloids, terpenoids, phenolics (including flavonoids), and phytoalexins (Jahan et al., 2025; Ali et al., 2024; Monsalvo et al., 2024; Cai et al., 2023; Yang et al., 2024a). Among them, plant antitoxins (phytoalexins) are specific metabolites synthesized *de novo* during infection, originating from the phenylpropanoid pathway, terpenoid or indole pathways (Wu et al., 2023; Muñoz-Hoyos and Stam, 2023; Yadav et al., 2020), and are not only able to kill pathogens directly, but also act as signaling molecules to amplify host immune response (Zhao et al., 2023; Zhan et al., 2022; Adhikary and Dasgupta, 2023).

It has been shown that erucamide synthesized by *Arabidopsis thaliana* under stress blocks the assembly of the bacterial T3SS needle protein SctF, thereby reducing pathogenicity and establishing a metabolite-based defense system (Miao et al., 2025). In addition, the volatile secondary metabolite citral was found to down-regulate several effector genes (e.g., *PcAvh137*, *PcAvh238*, *PcSCR5*) in *Phytophthora capsici*, effectively reducing its infectivity (Song et al., 2023). These findings highlight the dual role of secondary metabolites in plant disease resistance and insect defense. Meanwhile, in *Arabidopsis thaliana*, *AtWRKY33* promotes camalexin accumulation through the MAPK signaling pathway, and this metabolite not only enhances resistance to pathogens but also exerts an inhibitory effect on aphids (Zhou et al., 2022b; Kettles et al., 2013; Chen and Zhang, 2024b). In *Nicotiana attenuata*, *NaWRKY70* activates the transcription of *NaF6’H1*, a key gene in coumarin biosynthesis, thereby promoting the accumulation of scopoletin and its glycoside scopolin, which enhances resistance to *Alternaria alternata* (Song and Wu, 2024b, c; Sun et al., 2014). Studies in these model plants provide an important foundation for elucidating WRKY-regulated, secondary metabolite-mediated immune mechanisms.

In medicinal plants, the defensive function of WRKY TFs is closely linked to the metabolic regulation of their unique active components, reflecting an integration of immune defense and pharmacological value. For example, in *Withania somnifera*, WsWRKY1 enhances resistance to insect feeding by regulating withanolide accumulation and phytosterol-mediated defense pathways (Singh et al., 2017). In *Artemisia annua*, AaWRKY1 and AaWRKY17 positively regulate the expression of artemisinin-synthesising genes (*AaDBR2*, *AaCYP71AV1*, *AaADS*), thereby strengthening immune responses against *Pseudomonas syringae* pv. *tomato* DC3000; meanwhile, artemisinin exerts anti-malarial effects by disrupting *Plasmodium* proteins (Han et al., 2014; Zhan et al., 2023; Chen et al., 2021b). In *Taxus* spp., TcWRKY1, TcWRKY33, and TcWRKY26 activate key genes such as DBAT to promote paclitaxel accumulation, which shows antimicrobial activity *in vitro*, though its direct role in enhancing resistance in planta remains unconfirmed (Li et al., 2013; Chen et al., 2021c, 2022).

Phenolic and flavonoid compounds exhibit antimicrobial activity, reinforce cell walls, and induce systemic acquired resistance (SAR), a crucial component of sustained defense (Saini et al., 2024; Li et al., 2025c). For example, in medicinal plants including *Erigeron breviscapus* (Song et al., 2024d), *Passiflora edulis* (Ma et al., 2024), *Sophora flavescens* (Li et al., 2024d), and *Lycium barbarum* (Tong et al., 2025), multiple WRKY TFs (e.g., EbWRKY44, PeWRKY30, SfWRKY29, LcWRKY3, and LcWRKY13) positively regulate flavonoid accumulation, while others, such as PeWRKY12, may act as negative regulators to maintain immune homeostasis. PpWRKY70 activates the promoters of *4CL* and *PAL*, thereby increasing the synthesis of total phenolics, flavonoids, and lignin, and enhancing *Prunus persica* fruit resistance to *Rhizopus stolonifer*, highlighting the key regulatory role of WRKY TFs in the phenylalanine pathway and plant immunity (Ji et al., 2021).

In conclusion, WRKY TFs play a crucial role in enhancing the direct defense of medicinal plants against pathogens by regulating the synthesis of diverse classes of secondary metabolites, thus broadening the understanding of plant immune regulation. To further illustrate these regulatory relationships, we summarize the classification of WRKY-regulated secondary metabolites (**Table 2**).

**Table 2:** Classification of WRKY-regulated secondary metabolites.

Form	Name	Medicinal plant	WRKY	Machine	Bibliography
alkaloid	berberine	*Coptis chinensis* Franch	*CcWRKY7;* *CcWRKY29*; *CcWRKY32*	WRKY binds and activates the target gene *CcCNMT*	(Huang et al., 2023)
	tropane alkaloid	*Anisodus acutangulus*	*AaWRKY11*	Binds to and activates expression of the *AaH6H1* promoter and is involved in tropane alkaloid synthesis	(Zhou et al., 2024)
	camalexin	*Arabidopsis thaliana*	*AtWRKY33*	JA, ET regulation leads to camalexin accumulation and enhances pathogen resistance	(Zhou et al., 2020)
	camptothecin	*Ophiorrhiza pumila*	*OpWRKY2*	Activation of the core gene of the camptothecin pathway *OpTDC*	(Hao et al., 2021)
	Vincristine	*Catharanthus roseus *	*CrWRKY1*	Activation of *TDC* and *ZCT* genes	(Suttipanta et al., 2011)
	melatonin	*Manihot esculenta*	*MeWRKY20;* *MeWRKY75*	Increased *Manihot esculenta* melatonin levels 3-fold	(Wei et al., 2018)
	Benzylisoquinoline alkaloids	*Nelumbo nucifera*	*NnWRKY70a;* *NnWRKY70b*	Positive regulation of phenylethylamine alkaloids (BIAs) biosynthesis in response to jasmonic acid signaling	(Li et al., 2022)
	withanolide	*Withania somnifera*	*WsWRKY1*	Regulation of triterpenoid alkaloid withanolide accumulation	(Singh et al., 2017)
terpenoid	Tanshinones	*Salvia miltiorrhiza* Bunge	*SmWRKY2*	Up-regulation of the expression of the synthetic gene *SmCPS*	(Deng et al., 2019)
	artemisinin	*Artemisia annua*	*AaWRKY17*	Binding to the promoter of the artemisinin biosynthesis gene *ADS* against *Pseudomonas syringae*	(Chen et al., 2021b)
	*Saponins*	*Panax ginseng*	*PgWRKY4X*	Binds to the *PgSE* (squalene epoxidase) promoter and activates saponin synthesis	(Yao et al., 2020b)
	Patchoulol	*Pogostemon cablin* (Blanco) Benth	*PatWRKY71 *	Regulates Patchoulol biosynthesis	(Li et al., 2024e)
	sesquiterpene	*Aquilaria sinensis* (Lour.) Gilg	*AsWRKY44*	Inhibition of Sesquiterpene Biosynthesis Gene *ASS1* Transcription	(Sun et al., 2020)
	taxol	*Taxus*	*TcWRKY26*	Activates expression of the taxol biosynthesis gene *DBAT* to promote taxol synthesis	(Chen et al., 2022)
	ginsenoside	*Panax quinquefolius*	*PqWRKY1*	Involvement of MeJA in ginsenoside synthesis in response to MeJA	(Sun et al., 2013)
	carotenoid	*Solanum lycopersicum* L.	*SlWRKY35*	Activation of *SlDXS1* gene expression in the MEP pathway	(Yuan et al., 2022)
	monoterpene	*Litsea cubeba*	*LcWRKY17*	Binds to the promoter region of monoterpene synthesis-related genes (e.g. *TPS42*) and activates their expression	(Gao et al., 2023)
	β-ocimene	*Jasminum sambac*	*JsWRKY51*	Binding to the promoter region of genes related to aromatic terpene synthesis (e.g. *TPS*) activates their expression	(Lu et al., 2023)
phenolic compound	Lignin and flavonoids	*Nicotiana tabacum* L.	*NtWRKY28*	Regulation of Lignin and Flavonoids Synthesis Gene Expression Improves Defense Against M. persicae	(Chu et al., 2025)
	flavonoids	*Lycium ruthenicum* Murr.	*LrWRKY32*	Stimulation of *LrCYP75B1* expression, rutin synthesis	(Du et al., 2024)
	lignan	*Isatis indigotica*	*IiWRKY34*	Binding to the promoter region of *Ii4CL3*, a key rate-limiting enzyme gene for lignan synthesis	(Xiao et al., 2020)
	lignin	*Rosa* spp.	*RhWRKY30*	Activates the expression of *RhCAD1*, a key gene for lignin biosynthesis, promotes lignin accumulation, and enhances rose petal resistance to gray mold	(Li et al., 2024f)
	Proanthocyanidins	*Vitis quinquangularis*	*VqWRKY56*	Activation of PA biosynthetic genes for enhanced resistance to Powdery mildew pathogens	(Wang et al., 2023c)
	lignin	*Gossypium hirsutum*	*GhWRKY1-like*	Activation of *GhPAL6* and *GhCOMT1* expression positively regulates cotton resistance to *Verticillium dahliae*	(Hu et al., 2021)
	anthocyanins	*Malus domestica*	*MdWRKY40*	Interaction with MdMYB1 activates anthocyanins biosynthesis-related gene expression	(An et al., 2019)
	Acteoside	*Rehmannia glutinosa*	*RgWRKY37*	Activates the promoter activity of genes key to acteoside biosynthesis (e.g. *RgUGT* and *RgPAL*).	(Wang et al., 2021c)
	baicalin	*Scutellaria baicalensis* Georgi	*SbWRKY75;* *SbWRKY41*	Activation of JA signaling pathway to enhance baicalin biosynthesis	(Fang et al., 2023)

## 4 WRKY mediates epigenetic regulation of immune responses in medicinal plants

Notably, WRKY TFs regulate their own expression as well as downstream defense genes through epigenetic mechanisms such as DNA methylation and histone modifications, enabling precise control of immune responses.

When plants are attacked by pathogens, epigenetic modifications, including DNA methylation, histone acetylation, and histone methylation, alter the chromatin state of WRKY genes and their targets, thereby precisely regulating immune responses. Under pathogen-infected conditions, WRKY TFs bind to regulatory elements introduced by domesticated transposable elements (TEs) and modulate these elements through H3K27me3 modifications and DNA methylation, enabling Arabidopsis to activate precise immune responses during pathogen attack (Barco et al., 2019; Halter et al., 2021; Hure et al., 2025; Li et al., 2023). In addition, acetylation of histones H3 and H4, as well as H3K4 methylation in the WRKY promoter region, may facilitate transcriptional initiation of WRKY genes during pathogen infection (Jaskiewicz et al., 2011). Following Pseudomonas syringae infection of wild-type Arabidopsis, Trithorax 1 (ATX1) activates *WRKY70* by catalyzing trimethylation of histone H3 lysine 4 (H3K4me3), thereby enhancing SA signaling-mediated disease resistance (Alvarez-Venegas et al., 2007). Furthermore, Arabidopsis LDL1 and LDL2, homologous to human lysine demethylase 1-like 1, remodel chromatin accessibility by demethylating histone H3K4 at defense gene loci such as *WRKY22, WRKY40, and WRKY70*, thereby influencing the epigenetic regulation of plant immunity (Noh et al., 2021).

A growing body of evidence highlights the critical role of non-coding RNAs in plant immunity. For example, WRKY1 activates the expression of lncRNA33732, which in turn upregulates RBOH, leading to ROS, particularly H_2_O_2_ accumulation during the early immune response in tomato, thereby enhancing resistance to *Phytophthora infestans* (Cui et al., 2019). In rice, researchers identified a circular RNA named circ-WRKY9, which encodes a peptide of 88 amino acids (WRKY9-88aa). Overexpression of this peptide not only effectively inhibits rice stripe mosaic virus (RSMV) infection but also enhances immunity against rice blast and bacterial leaf blight (Pan et al., 2025).

## 5 Outlook

Medicinal plants harbor diverse bioactive compounds and exhibit strong responsiveness to environmental fluctuations and pathogen attacks. Diseases not only reduce plant growth and yield but also directly compromise the stability and quality of medicinal compounds. In recent years, the integration of CRISPR/Cas gene editing and synthetic biology with high-throughput transcriptomics, proteomics, and metabolomics has accelerated research on immune networks and key TFs in medicinal plants, offering novel theoretical frameworks and technical tools to improve disease resistance.

Existing studies have identified some immune regulatory modules through histological analyses. However, significant challenges remain, including unclear mechanisms and a disconnect between basic research and practical applications, making the transition to molecular breeding difficult. In the future, the integration of artificial intelligence and biotechnology is expected to overcome this bottleneck by enabling functional prediction of key immune genes, regulatory network modeling, and intelligent screening of the superior germplasm, thereby establishing a highly efficient and smart disease-resistant breeding system. By reconstructing transcription factor regulatory networks and optimizing signaling pathways, disease resistance in medicinal plants can be significantly enhanced, providing a solid foundation for the high-quality and sustainable development of the Chinese herbal medicine industry.

In conclusion, systematic analyses of key TFs’ immune functions in medicinal plants, integrated with multi-omics, gene editing, and artificial intelligence approaches, are anticipated to bridge the gap between basic research and breeding applications, thus facilitating the synergistic advancement of disease resistance research and the breeding of superior medicinal plant cultivars.

### 5.1 Molecular design breeding to accelerate transformation

To enhance the immunity of medicinal plants, immune-related factors can be heterologously expressed, overexpressed, or suppressed using transgenic breeding approaches utilizing advanced genetic transformation technologies, the Cut-Dip-Budding (CDB) technique. Such approaches not only confer desirable genetic traits to medicinal plants, facilitating gene function elucidation and targeted trait improvement, but also improve plant yield and enhance tolerance to pathogen infestation (Yan et al., 2022).

In transgenic research, commonly employed biological transformation methods include Agrobacterium-mediated and virus-mediated approaches. For instance, transferring WRKY disease resistance genes into medicinal plants through Agrobacterium-mediated transformation has been shown to effectively enhance their pathogen resistance. In papaya, overexpression of CpWRKY50 via Agrobacterium infiltration positively regulates anthracnose resistance by promoting JA signaling (Yang et al., 2024b). Similarly, Agrobacterium-mediated transformation of CsWRKY48 into tobacco enhanced its resistance to aphids (Wang et al., 2024b).

However, traditional genetic transformation methods are restricted to a limited number of medicinal plants and are often time-consuming. To overcome this limitation, the improved CDB technique was developed, allowing direct infection of medicinal plant organs, including the roots of *Taraxacum mongolicum* and *Rehmannia*, as well as the petiole of *Salvia miltiorrhiza*. This method not only enhances transformation efficiency but also prevents the formation of callus tissue and hairy roots (Cao et al., 2023, 2024). Through this approach, disease resistance-related genes can be efficiently delivered into medicinal plants, thereby improving their resistance to pathogens.

It is noteworthy that current genetic transformation systems are being continuously improved through RNA interference (RNAi) and gene editing technologies. The integration of these technologies with artificial intelligence applications can substantially improve the precision and efficiency of gene editing.

### 5.2 Artificial intelligence breakthroughs in medicinal plant immune networks

With the integration of gene editing and AI, research on medicinal plant breeding and immunity is entering a new phase of empirically driven innovation. AI has shown significant value across multiple key processes: from AlphaFold’s high-precision protein structure prediction, which enables the analysis of immune-related factors and the design of target sites (Ma et al., 2022), to novel tools such as CRISOT and CCLMoff that advance sgRNA optimization and off-target control. Collectively, these developments outline a promising technological pathway for achieving precise immunoediting in medicinal plants (Du et al., 2025; Chen et al., 2023; Lee, 2023).

In disease monitoring, AI-driven image recognition and environmental modeling are advancing rapidly. Experimental evidence shows that near-infrared and hyperspectral imaging provide high sensitivity and accuracy for early disease detection (Upadhyay et al., 2025). In addition, models based on transfer learning, such as You Only Look Once version 7 (YOLOv7) and version 8 (YOLOv8), can identify a wide range of diseases including powdery mildew, leaf spot and grey mold, and perform well on key metrics (mean accuracy mAP ≈ 91%, precision, recall, and F1 scores), underlining the potential of deep learning for fast and accurate identification. (YOLOv7) and version 8 (YOLOv8) can identify multiple diseases including powdery mildew, leaf spot, and grey mould, and perform well on key metrics (Mean Average Precision, mAP ≈ 91%; Precision; Recall; and F1-score), highlighting the potential of deep learning for fast and accurate identification (Sambana et al., 2025). These advances lay a foundation for dynamic monitoring and precise intervention in the immune networks of medicinal plants, and open possibilities for establishing a closed-loop system of monitoring, intervention, and verification to enhance disease resistance and ensure the stability of medicinal compounds.

Further, integrated prediction of genome and environment (iGEP), combining multi-omics data with machine learning, can optimize plant design at both macro and micro levels while capturing nonlinear features of high-dimensional data, thereby enabling accurate prediction of disease resistance mechanisms (Xu et al., 2022; Mohamedikbal et al., 2025). Although its application is still in the early stages, it has already provided important theoretical and technological support for AI-driven immune networks and “on-demand editing”.

Overall, integrating AI with multi-omics is shifting medicinal plant immunity research from passive resistance to proactive regulation, laying the foundation for intelligent and efficient medicinal plant breeding.
